# Neuroimaging of Human Balance Control: A Systematic Review

**DOI:** 10.3389/fnhum.2017.00170

**Published:** 2017-04-10

**Authors:** Ellen Wittenberg, Jessica Thompson, Chang S. Nam, Jason R. Franz

**Affiliations:** ^1^Edward P. Fitts Department of Industrial and Systems Engineering, North Carolina State UniversityRaleigh, NC, USA; ^2^Joint Department of Biomedical Engineering, University of North Carolina at Chapel Hill and North Carolina State UniversityChapel Hill, NC, USA

**Keywords:** static and dynamic balance control, temporal and spatial dynamics of brain activation, mechanical perturbation, sensory degradation, susceptibility to cognitive dual tasks, movement artifacts

## Abstract

This review examined 83 articles using neuroimaging modalities to investigate the neural correlates underlying static and dynamic human balance control, with aims to support future mobile neuroimaging research in the balance control domain. Furthermore, this review analyzed the mobility of the neuroimaging hardware and research paradigms as well as the analytical methodology to identify and remove movement artifact in the acquired brain signal. We found that the majority of static balance control tasks utilized mechanical perturbations to invoke feet-in-place responses (27 out of 38 studies), while cognitive dual-task conditions were commonly used to challenge balance in dynamic balance control tasks (20 out of 32 studies). While frequency analysis and event related potential characteristics supported enhanced brain activation during static balance control, that in dynamic balance control studies was supported by spatial and frequency analysis. Twenty-three of the 50 studies utilizing EEG utilized independent component analysis to remove movement artifacts from the acquired brain signals. Lastly, only eight studies used truly mobile neuroimaging hardware systems. This review provides evidence to support an increase in brain activation in balance control tasks, regardless of mechanical, cognitive, or sensory challenges. Furthermore, the current body of literature demonstrates the use of advanced signal processing methodologies to analyze brain activity during movement. However, the static nature of neuroimaging hardware and conventional balance control paradigms prevent full mobility and limit our knowledge of neural mechanisms underlying balance control.

## Introduction

Approximately 30% of adults aged 65 years or older experience one or more falls annually, a third of which result in a moderate to severe injury (Alexander et al., 1992). In addition to the high financial burden associated with these falls, there is also a loss of independence and increased risk of mortality (Brauer et al., 2000). With our aging population, the incidence of falls will likely continue to rise. Slips and falls are usually due extrinsic, environmental factors, including surface contamination, lighting, and shoe-type. However, intrinsic factors also contribute to falls, including age, pathologies, medications, attention, fatigue, and physical status (Gauchard et al., 2001). Loss of static balance control, such as can occur when standing on a moving bus, and loss of dynamic balance control, such as can occur when walking in a dark room, both contribute to slips and falls.

Static and dynamic human balance control, and changes thereof due to intrinsic and extrinsic factors, have garnered considerable scientific and clinical attention. Walking balance control is especially dynamic, involving coordinated adjustments in posture (i.e., head and trunk stabilization) and foot placement from step to step (Bauby and Kuo, 2000; Kay and Warren, 2001; Donelan et al., 2004; Rankin et al., 2014). Particularly in unpredictable and challenging environmental conditions, these adjustments depend on the integration of reliable sensory feedback and the planning and execution of appropriate motor responses (O'Connor and Kuo, 2009; O'Connor et al., 2012; Francis et al., 2015; Franz et al., 2015, 2016; Goodworth et al., 2015). Accordingly, sensory and mechanical perturbations are increasingly used to study corrective motor responses in standing and walking and the onset and progression of balance deficits. Sensory perturbations may include those of visual (e.g., optical flow) (O'Connor and Kuo, 2009; O'Connor et al., 2012; Francis et al., 2015; Franz et al., 2015, 2016), somatosensory (e.g., tendon vibration) (Gurfinkel et al., 1976; Hay et al., 1996; Bove et al., 2003; Mullie and Duclos, 2014), or vestibular feedback (e.g., galvanic stimulation) (Day et al., 1993; Fitzpatrick et al., 1994; Bent et al., 2002; Dakin et al., 2007; Dalton et al., 2014), whereas mechanical perturbations most frequently incorporate support surface translations (Sinitksi et al., 2012; Aprigliano et al., 2016). Cortical activity and high-order cognitive processes are highly involved in the planning and execution of these motor responses. Indeed, dual tasks during standing and walking elicit cognitive-motor interference that compromise metrics of balance control (Dubost et al., 2006; Priest et al., 2008; Plummer et al., 2015).

Human balance control investigations have primarily focused on quantifying motor responses based on kinematic measurements such as movement variability and dynamic stability and/or kinetic measurements such as center of pressure and angular momentum (O'Connor and Kuo, 2009; McAndrew et al., 2011; Francis et al., 2015; Sheehan et al., 2015). These studies are rapidly accelerating our scientific understanding of human balance control, with exciting translational implications for the diagnosis and rehabilitation of people at risk of falls. However, this promising translational potential is currently limited by our relatively incomplete understanding of central mechanisms involved in human balance control and ultimately changes thereof due to aging or disease. Advancements in the use of neuroimaging, and in particular the development of advanced mobile measurements, are now providing previously inaccessible insight into brain connectivity during functional movement and balance tasks.

## Review objectives

Evidence from neuroimaging studies reveals cortical involvement in standing and walking, with modalities having high spatial resolution, such as functional magnetic resonance imaging (fMRI), single-photon emission computed tomography (SPECT), and positron emission tomography (PET), leading to the identification of a supraspinal locomotor network (la Fougere et al., 2010; Zwergal et al., 2013). Within this supraspinal network, la Fougere et al. (2010) identified a direct locomotor pathway, used primarily during gait execution, and the indirect locomotor pathway, activated during gait planning (see Table 1). The main difference between the pathways involves activating the pre and post central gyri in the direct pathway in contrast to activating the dorsolateral prefrontal cortex (DLPFC) and basal ganglia in the indirect pathway. Furthermore, Zwergal et al. (2013) and others have observed activation of brain regions of the indirect locomotor pathway gait execution in populations with neurological disorders.

**Table 1:** **Motor pathways in la Fougere et al. (2010)**.

	**Direct locomotor pathway**	**Indirect locomotor pathway**
Brain activation	•Pre and post central gyrus (BA 3, 4) •Lingual and fusiform gyrus (BA 19, 37) •Parahippocampal gyrus (BA 36, 27) •Cuneus and precuneus (BA 18, 31) •Insula and inferior frontal gyrus (BA 13, 47) •Cerebellum and brainstem (vermis, paravermis, pontine tegmentum)	•Middle frontal gyrus (BA 6) •Superior frontal gyrus (BA 9,10) •Parahippocampal gyrus (BA 19, 36) •Precuneus (BA 7, 31) •Middle occipital gyrus (BA 18) •Cingulate gyrus (BA 32, 24) •Anterior insula (BA 13) •Superior temporal gyrus (BA 22) •Supramarginal gyrus (BA 40) •Putament, caudate nucleus, cerebellum, and brainstem (pontine tegmentum)
Brain deactivation	•Inferior temporal gyrus (BA 20) •Inferior parietal lobe (BA 29) •Frontal and medial frontal gyrus (BA 6) •ACC (BA 32)	•Superior temporal gyrus (BA 22)

*BA represents the Brodmann Area*.

Neuroimaging studies utilizing functional near-infrared spectroscopy (fNIRS) have allowed for acquisition of brain activity during upright stance and with some degree of subject mobility. Using fNIRS, differential activation of the sensorimotor cortices, supplementary motor areas, and prefrontal cortex have been observed during gait initiation (i.e., standing to walking) and steady-state walking (Miyai et al., 2001; Suzuki et al., 2004; Mihara et al., 2012). Activation of the prefrontal cortex and supplementary motor areas have been correlated with variations in step-width and stride-time intervals (Kurz et al., 2012; Caliandro et al., 2015). Although the increased mobility of fNIRS has allowed for neuroimaging during real gait, research is still limited by poor temporal resolution. The use of electroencephalography (EEG) allows for higher temporal resolution and has potential for mobile applications (Jeon et al., 2011; Nam et al., 2011, 2012; Li and Nam, 2016). Advances in hardware systems have allowed for free movement in space, with wireless data transmission. Although the hardware is mobile, acquiring brain signals during movement introduces signal artifacts that compromise the integrity of underlying electrocortical activity (Gwin et al., 2010). Identifying and removing these artifacts has been challenging; however, recent advances in both hardware and signal analysis techniques show promise in developing fully mobile EEG neuroimaging systems (Gramann et al., 2014).

Understanding the neural underpinnings of human balance control is an essential next step in addressing slip and fall risks in the general population. While spatial brain activation during steady-state walking has been reviewed by Hamacher et al. (2015), to our knowledge, there is no current review analyzing changes in brain activity due to static and dynamic balance control challenges, including spatial, temporal, and frequency analyses. Additionally, the impact of mobile neuroimaging hardware paired with non-restrictive balance control tasks has not yet been evaluated. Therefore, this study reviewed the current neuroimaging literature investigating the neural correlates of human balance control to address the following three research questions:

- **Research Question (RQ) 1:** What are the spatial and temporal dynamics of brain activity when mechanical perturbations, cognitive tasks, and modulation of sensory inputs challenge static balance control?
  Evaluation of human balance control uses a variety of paradigms to challenge motor, cognitive, and sensory components of balance (de Oliveira et al., 2008). Animal models, lesion studies, and neuroimaging evidence support cortical involvement in maintaining upright stance due to external perturbations (Jacobs and Horak, 2007). However, the spatial and temporal characteristics of brain activity evoked in varying balance control paradigms has yet to be analyzed.
- **Research Question (RQ) 2:** What are the spatial and temporal dynamics of brain activity when mechanical challenges, cognitive tasks, and modulation of sensory inputs test dynamic balance control?
  While brain activity during walking has been recently reviewed (Hamacher et al., 2015), there is no current analysis on the impact of balance challenges on dynamic balance control.
- **Research Question (RQ) 3:** What methods have been used to identify and remove movement artifact from brain signals acquired during balance control tasks?

Neuroimaging modalities are sensitive to head and body movement, which introduces movement artifact into the acquired brain signal. The characteristics of movement artifact are related to both the neuroimaging modality and the research paradigm, with minimal artifact when subjects are completely still. However, it is reasonable to expect sudden movements to maintain static balance control or rhythmic walking during dynamic balance control to introduce variable movement artifact. In order to identify neural mechanisms involved in balance control, movement artifact must be correctly identified and removed. Therefore, it is important to identify the artifact removal methods used in static and dynamic balance control studies.

## Review method

### Search strategy

**Figure 1:**
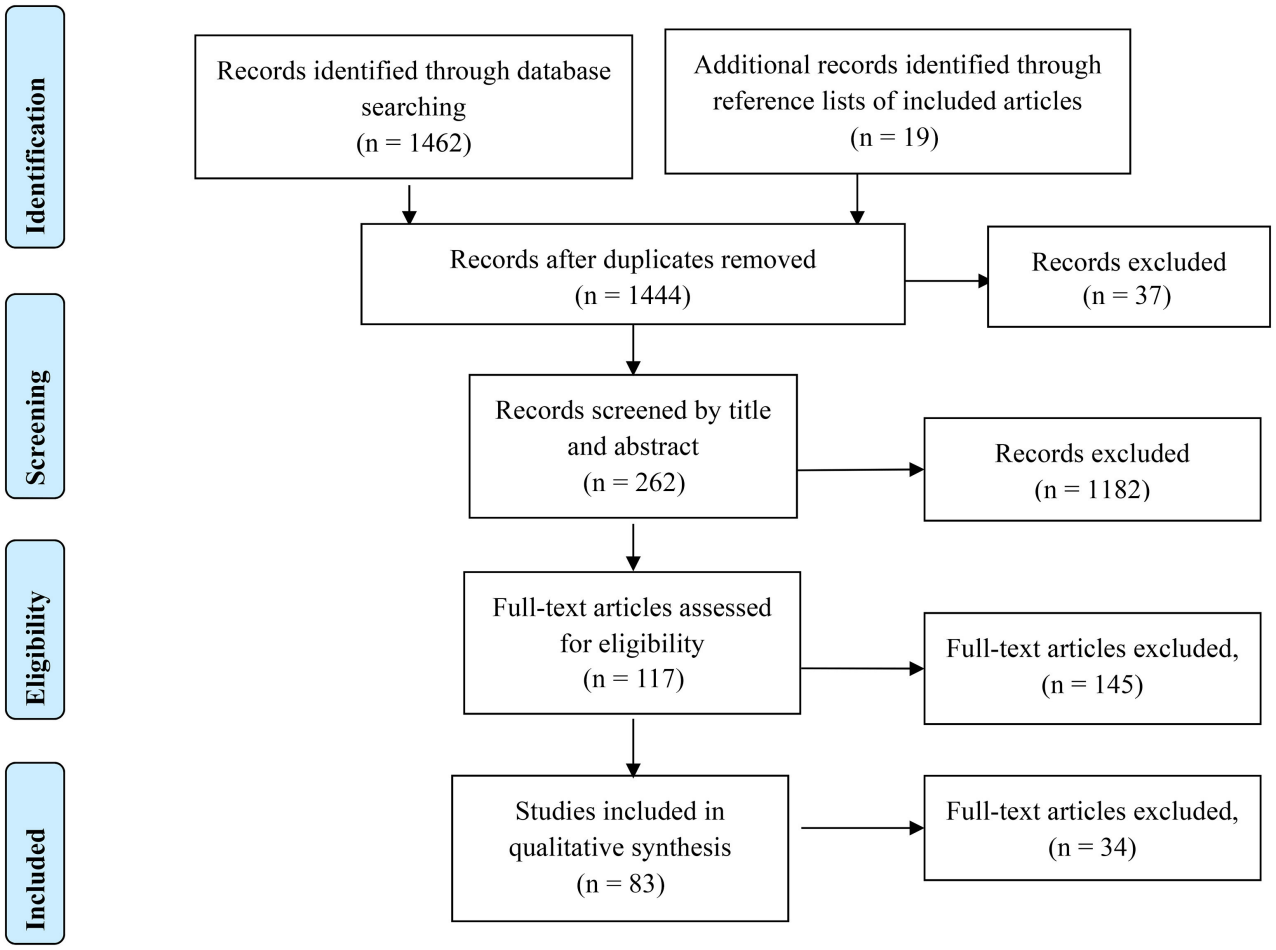
**PRISMA flow diagram of this review**.

This systematic review utilized the Preferred Reporting Items for Systematic Reviews and Meta-Analyses (PRISMA; see Figure 1) approach (Liberati et al., 2009). The searches included detailed terms related to neuroimaging, static and dynamic balance control, and brain activity. The three search fields were connected with “AND” to ensure at least one term of each field could be found in the results. The terms in each of search field were linked with “OR.”

### Search terms

Neuroimaging search terms included variations of the following: fMRI, EEG, fNIRS, MEG, PET, and SPECT. Balance control search terms included variations of the following: Standing, balance, posture, gait, stepping, walking, plantarflexion, dorsiflexion, locomotion, and postural control. Brain activity search terms included variations of the following: Cortical activity, subcortical activity, neural activity, executive function.

### Search process

The primary information sources included in this review are: (1) IEEExplore and (2) Compendex, both to provide an engineering perspective, (3) ACM Library, to provide a computing and signal processing perspective, (4) PubMed, to provide a clinical perspective, and (5) Web of Science, to provide a cross disciplinary perspective. The database search included search terms found in the article title, abstracts, and keywords. The results from each database were added to Mendeley and checked for any duplicate results.

### Inclusion and exclusion criteria

Search inclusion and exclusion were based on the characteristics and goals of the balance control tasks and the use and application of neuroimaging modalities. Literature included in this review aimed to investigate human balance control. Studies were included if they incorporated balance challenges (e.g., perturbations, eyes closed, dual-task, etc.). Only articles in English were considered. We excluded literature from this review when either the balance control paradigm or the neuroimaging paradigm did not align with the goals of this study. Studies that evaluate the effects of a therapy or intervention, including drug or hormone trials, rehabilitation, training, robotic-assisted walking, or development (in children and adolescents) were excluded. Studies that did not include active subject movement, such as those using motor imagery, brain volume correlation, or passive walking paradigms were excluded. Passive walking paradigms are those where the experimenter or specially designed equipment assisted the subject in moving their legs (Dobkin et al., 2004). Additionally, studies that use isolated joint movement and coordinated body movement (arms and legs) were excluded. Lastly, studies that aimed to evaluate technological advancements with no concurrent evaluation of human subjects were excluded.

### Data extraction

After agreeing on studies to be included, the entirety of each article was examined, and studies were divided into two categories: (1) brain activity during static balance control tasks and (2) brain activity during dynamic balance control tasks. Studies in both groups that included methodologies for movement artifact identification and removal were pooled to address research question three. The subsequent data were then extracted from each article: Authors, year of publication, static or dynamic balance task, type of balance challenge (further categorized as sensory, mechanical or cognitive), treadmill or overground walking (for dynamic studies), subject characteristics, modality of brain imaging, and brain activity (including spatial, temporal, and/or frequency response). As well as extraction of pre-processing, spatial filtering, and artifact removal methods.

## Results and discussions

### Study selection and characteristics

Literature searches included relevant studies published on or before September 1, 2016, and resulted in 1,462 results, with 1,444 studies remaining after duplicate removal. After initial screening of the abstract, 117 studies were evaluated for inclusion in the static balance control or dynamic balance control category. The full-text articles for each of the studies was reviewed, a total of 38 studies were eligible for inclusion in the static balance control group and 47 for the dynamic group, with two studies including both static and dynamic balance control tasks. All 83 studies included in the static and dynamic groups were evaluated for inclusion in addressing research question three regarding identification and removal of movement artifact. Figure 1 shows the PRISMA approach used in the present study.

### Studies selected for RQ1

This review covered 38 studies investigating neural correlates of static balance control. The balance challenge paradigms include mechanical perturbations, cognitive, dual-task paradigms and sensory degradation, or impairment, which can be found in Tables 2–**4**, respectively.

**Table 2:** **Brain activity due to mechanical challenges to static balance control**.

**Name, year**	**Balance challenge**	**Modality**	**Mobile**	**Spatial location**	**Activity**
Adkin et al., 2008	Please refer to Table 3				
Adkin et al., 2006	Single transient horizontal perturbations to the trunk	EEG	No	Cz	N100 amplitude
Bulea et al., 2014	Sit-to-stand transitions	EEG	Yes	Frontal cortex, motor strip, parietal cortex, and central midline regions of interest	Alpha and theta band power greater at rest than pre-movement
					Higher delta band power pre- and post-movement vs. rest
					Classification of lower extremity movement intent based on pre-movement delta band signal
Chang et al., 2016	Please refer to Table 4				
Del Percio et al., 2009	Unipedal vs. bipedal stance	EEG	No	Lt and rt central, rt. and middle parietal	Amplitude of alpha ERD
				Rt frontal, central, middle parietal	Amplitude of alpha ERD
Huang et al., 2014	Please refer to Table 3				
Hülsdünker et al., 2016	Unstable surface conditions via platform unexpected perturbations	EEG	No	Frontal, fronto-central, and fronto-parietal	Alpha band power
				Midline	Theta band power
Hülsdünker et al., 2015	Bipedal vs. unipedal with various levels of support surfaces	EEG	No	Frontal, Central, Parietal	Increased theta power
				Fronto-central, fronto-parietal	Theta power
Jacobs et al., 2008	Unexpected vs. expected translation of platform	EEG	No	Cz, Pz, Fz, F3, F4	CNV
Marlin et al., 2014	Stand lean and release task and Flanker task	EEG	No	ACC	ERN Flanker task response
				Medial frontal gyrus and supplementary motor area	N100 evoked by perturbations
Mierau et al., 2015	Horizontal perturbations of platform	EEG	No	Localization in parietal cortex	P100 evoked by perturbations
				Localization in midline fronto-central cortex	N100 evoked by perturbations
Mihara et al., 2008	Horizontal perturbations of platform	fNIRS	No	PFC, DLPFC	Activation after external perturbation
				Rt. Posterior parietal cortex and SMA	Increased activation
Mihara et al., 2012	Horizontal translations of platform in older, hemiplegic stroke patients	fNIRS	No	PFC, premotor and parietal areas	Increased activation due to perturbation
				SMA and PFC	Activation
Mochizuki et al., 2008	Unpredictable perturbations to the support surface	EEG	No	Cz	N100 post-perturbation amplitude
Mochizuki et al., 2009b	Perturbations that were cued externally or self-initiated	EEG	No	Cz, FCz, Fz, CPz, C1, C2, C3, and C4	N100 post-perturbation amplitude
Mochizuki et al., 2009a	Mechanical postural perturbations during sitting and standing	EEG	No	FCz	Instability evoked N100
				CPz	Amplitude of instability evoked P200
Ouchi et al., 1999	Please refer to Table 4				
Petrofsky and Khowailed, 2014	Please refer to Table 4				
Quant et al., 2005	Horizontal translations of platform with varying deceleration	EEG	No	Cz	N200 and P200 amplitude and latency
Slobounov et al., 2009	Single-legged, eyes-closed balance task to study pre-falling, and transition to instability	EEG	No	ACC, precuneus, parietal lobe, and occipital cortex	Theta, alpha, and gamma bands
Slobounov et al., 2008	Voluntary postural sway in the AP and ML directions	EEG	No	Frontal, Fronto-central, parietal	Alpha, beta, gamma power
					Amplitude of MRCP
Slobounov et al., 2005	Oscillatory swaying motions in the from the ankle	EEG	No	Frontal, Central	High gamma band
				Frontal, Central, Parietal	Presence of MRCP
Smith et al., 2012	Backward surface translations in older adults with or without PD	EEG	No	Cz	Beta band ERD
				Cz	Amplitude of CNV
Smith et al., 2014	Mechanical perturbations while cued to perform maximal postural response in older, PD patients	EEG	No	Cp1, Cz	Alpha, Beta ERD
				Cz	Amplitude CNV
Tse et al., 2013	Please refer to Table 4				
Varghese et al., 2015	Please refer to Table 4				
Varghese et al., 2014	Lean and release cable system	EEG	No	FCz	Perturbation evoked N100 amplitude

*PD, Parkinson's Disease; lt., left; rt., right; bi. lat., bilateral; PFC, prefrontal cortex; DLPFC, dorsolateral prefrontal cortex; SMA, supplemental motor area; ACC, anterior cingulate cortex; ERD, event related desynchronization; CNV, contingent negative variation; ERN, error related negativity potential; MRCP, movement related cortical potential*.

#### Mechanical challenges

Twenty-seven studies used mechanical perturbations to elicit a balance control response, 15 of which focused on the evoked cortical potentials (See Table 2). The negative potential occurring around 100 ms following an event, such as mechanical perturbations, is termed the N100 potential. The N100 response over the fronto-central area has been observed in a wide range of balance tasks, and N100 amplitude increases in challenging balance control tasks, including unpredictable or surprise perturbations, and in balance challenges with low sensory inputs (Adkin et al., 2006, 2008; Mochizuki et al., 2008; Huang et al., 2014; Varghese et al., 2014, 2015). However, Mochizuki et al. (2009a) observed no difference in N100 latency and amplitude in sitting and standing instability conditions, suggesting that there may be more general processes that underlie stability, regardless of sensory, motor, or postural aspects of response.

N100 is also called the error related negativity potential (ERN), which is known to be evoked when an error is committed and to originate in the anterior cingulate cortex (ACC). Marlin et al. (2014) investigated if the perturbation evoked N100 and the ERN both originated in the ACC using a lean-and-release protocol to invoke a feet-in-place balance response [similar to those in: Adkin et al. (2006) and Mochizuki et al. (2008)] and used dipole analysis to locate the N100 response. The ERN was localized to the ACC, as expected. However, the perturbation related N100 was localized to the medial frontal gyrus and supplementary motor area. These results indicate that perturbation evoked N100 is related to motor processes rather than a general error event.

Although there has been research in the early cortical response following perturbations, the role of later potentials is still unclear. In the auditory domain, for example, the event related potential (ERP) component around 200 ms post-stimulus (P200), represents a shift in attention toward the initial audio cue. Quant et al. (2005) used horizontal platform translations with quick or delayed deceleration to determine if P200 in the balance domain was related to a shift in attention or indicated sensory or motor processes. There was no difference in N200 and P200 amplitude or latency in immediate vs. delayed decelerations, suggesting that the motor reactions needed to maintain stability and later cortical responses are likely independent (Quant et al., 2005). Another event related potential is the contingent negative variation (CNV), a slow potential related to anticipatory attention, preparation, and motivation (Nagai et al., 2004). The CNV has been observed in response to unexpected surface perturbations in the midline and frontal areas (Jacobs et al., 2008) and to perturbations with an unexpected magnitude at Cz (Smith et al., 2012). Subjects in these studies are preparing for an unexpected surface perturbation, which explains the CNV response. However, Smith et al. (2014) provided amplitude cuing of the upcoming response in Parkinson's Disease patients and did not observe a CNV response. Given that subjects successfully demonstrated balance control, these results indicate that another type of cortical response is involved in cued responses.

Thirteen EEG studies performed frequency analysis. As task difficulty increased (for example, decreased surface support due to standing on foam), alpha power in healthy controls decreased, indicating increased cortical activation (Petrofsky and Khowailed, 2014; Hülsdünker et al., 2016). Slobounov et al. (2008) observed a larger decrease in alpha power in central electrodes prior to sway in the medio-lateral direction compared to an anterior-posterior sway. While performance studies have found anterior-posterior sway magnitude and torque is larger than medio-lateral sway, these results suggest that cortical activity may be sway-direction dependent, with medio-lateral instability requiring more cortical control in self-initiated postural movements. Lastly, Slobounov et al. (2009) saw a drop in alpha power in occipital region prior to a fall. However, when subjected to the challenging balance conditions, trans-tibial amputees exhibited an increase in alpha power (Petrofsky and Khowailed, 2014). Chang et al. (2016) challenged balance control of older adults in high and low fall risk groups by subjecting them to anterior-posterior motion via a Steward Platform. Two conditions were tested with and without a virtual-reality based scenery synchronized with the motion platform. Beta band power increased in the virtual-reality condition in the frontal and occipital regions for both groups. Petrofsky and Khowailed (2014) also observed an increase in beta power as balance challenges increased in central and parietal areas of both controls and amputee patients. Tse et al. (2013) also found increase in beta power with increase in balance challenge task (base of support or surface compliance) in the parietal and central areas. Smith et al. (2012) observed an increased in beta band event related desynchronization (ERD) at Cz for predictability predictable, small magnitude perturbations. However, in a similar paradigm with and without amplitude cuing, Smith et al. (2014) did not observe a significant difference in beta power between the two conditions. In comparing the demands of balance during medio-lateral and anterior-posterior sway, Slobounov et al. (2008) found that beta power in the central region dropped significantly more prior a self-initiated ML sway.

Recent studies have also investigated theta band increases in challenging balance tasks in frontal, central, and parietal areas (Hülsdünker et al., 2015, 2016; Chang et al., 2016). Hülsdünker et al. (2015) found that theta power over the fronto-central and central-parietal areas correlated with balance performance. Slobounov et al. (2009) found a midline theta burst during the shift from the stable stage to transition-to-instability stage which was localized to the ACC. This burst was followed by the reduction of theta power just preceding an actual fall. Lastly, gamma activation in various areas of the brain have been shown to increase with increased balance challenge (Tse et al., 2013; Chang et al., 2016). More specifically, gamma activity increased right before the initiation of a balance reaction in the frontal and central areas (Slobounov et al., 2005, 2008). These findings indicate that theta increases correspond with the timing of balance control reactions. Bulea et al. (2014) also found that the low frequencies of the EEG signal contain information regarding lower extremity movement and balance control. Utilizing the delta band frequencies from EEG signal acquired while subjects transitioned from sitting to standing posture, they were able to classify movement intent (i.e., if the subject was going to stand-up, sit-down, or remain at rest).

While neuroimaging analysis following EEG signal acquisition has high temporal resolution, it lacks spatial resolution. Therefore, PET and fNIRS have been used to investigate the spatial characteristics of a balance response. Ouchi et al. (1999) observed the hemodynamic response following bipedal or unipedal stance with eyes open or closed using PET, finding an increased activation of the cerebellar anterior vermis and posterior lobe lateral cortex during unipedal stance and increased activation of the cerebellar anterior lobe and right visual cortex during bipedal stance. The visual cortex and vermis were activated during standing with feet together with eyes on a target as well (Ouchi et al., 1999). These findings provide spatial insights into neural correlates of balance control, mainly that the cerebellar vermis and visual cortex may be involved in maintaining and regulating standing posture. Studies using fNIRS also reveal cortical involvement in balance control. Mihara et al. (2008) observed an activation of the prefrontal cortex (PFC) and dorsolateral prefrontal cortex (DLPFC) after anterior-posterior and medio-lateral horizontal perturbations accompanied by increased activation of the right posterior parietal cortex and supplementary motor area in conditions with auditory warning signals. Similarly, Mihara et al. (2012) observed activation of prefrontal cortex, premotor, and parietal areas following anterior-posterior and medio-lateral horizontal perturbations in older, hemiplegic stroke patients. These findings point to involvement of the prefrontal, premotor, supplementary motor, and parietal cortex in standing balance control.

#### Cognitive challenges

Seven studies required subjects to maintain balance while performing a cognitive dual-task paradigm (See Table 3). Dual-task paradigms necessitate allocation of attentional resources to perform both the cognitive and balance tasks, and performance outcomes have correlated with the integrity of balance control.

**Table 3:** **Brain activity due to cognitive challenges to static balance control**.

**Name, year**	**Balance challenge**	**Modality**	**Mobile**	**Spatial location**	**Activity**
Adkin et al., 2008	Perturbation under postural threat	EEG	No	Cz	Perturbation evoked N100 amplitude
Fujita et al., 2016	Stroop task during bipedal vs. unipedal standing	fNIRS	No	Rt. DLPFC	Increased activation
Huang et al., 2014	Tilt platform using visual feedback	EEG	No	Bi. lat. fronto-central and contralateral sensorimotor areas	Latency and amplitude of N100 for postural control
				Bi. lat. fronto-central and ipsilateral temporal areas	Latency and amplitude of P200 for postural control
				Lt. frontal-central area	MRP for postural control
Lau et al., 2014	Visual oddball response task while standing or walking	EEG	No	Sensorimotor cortex	Effective connectivity
				PFC, posterior parietal cortex, ACC	Effective connectivity
Little and Woollacott, 2015	Visual WM capacity during surface perturbations and walking	EEG	No	Lt. pre-motor and rt. sensory areas	Amplitude of N100 ERP
Mirelman et al., 2014	Walking while counting forward, walking with serial 7's and serial 7's in standing	fNIRS	No	Fp1 and Fp2	Increased activation with increased task difficulty
Quant et al., 2004	Horizontal translations platform with or without a visuomotor track task	EEG	No	Cz	Amplitude of perturbation evoked N100

*lt., left; rt., right; bi. lat., bilateral; PFC, prefrontal cortex; ACC, anterior cingulate cortex; MRP, movement related potential*.

A decrease in N100 amplitude was observed in visual working memory task and visuo-motor track task conditions (Quant et al., 2004; Little and Woollacott, 2015). Similarly, Huang et al. (2014) provided subjects with visual feedback to facilitate maintaining balance on a tilt platform, resulting in decreased N100 amplitude over the motor cortex and sensorimotor areas. These studies found a decrease in N100 in the dual-task conditions, indicating their efficacy to split attentional resources between balance and cognitive tasks. In contrast, Adkin et al. (2008) evoked an emotional response (fear; anxiety) by placing subjects at a prescribed height off the ground. An N100 response was observed prior to an unpredictable perturbation, and N100 amplitude increased with increasing height. Mirelman et al. (2014) had subjects stand still and perform serial seven subtractions, a common cognitive loading task, resulting in increased activation in the left and right frontal lobe compared to single-task standing. Fujita et al. (2016) also observed increased brain activity in a cognitive DT paradigm using fNIRS. High and low memory span groups performed one or two leg standing with a Stroop task. Increased right dorsolateral prefrontal cortex activation was observed in the high span group in both one and two leg standing dual-task conditions, compared to the low span group. Also, performance in both groups decreased during one leg, dual-task standing. Lastly, Lau et al. (2014) compared effective connectivity during standing or walking with or without a visual oddball discrimination response task. They found that connectivity was weaker during standing when performing a cognitive task in the prefrontal cortex, posterior parietal cortex, and ACC. However, effective connectivity was stronger in the standing conditions compared to the walking condition regardless of cognitive task. These findings suggest that more cognitive resources may be required to maintain standing posture, as compared to walking (Lau et al., 2014).

#### Sensory challenges

Experimentally manipulating sensory inputs (vestibular, visual, or proprioceptive) is another method to challenge balance control. Eleven studies used a form of sensory removal or augmentation, including eyes closed conditions and the use of virtual reality (See Table 4). For example, subjects stood with eyes closed and feet together or in tandem (i.e., heel-to-toe), which is more challenging, and N100 was evoked prior to the need for a balance reaction. The N100 amplitude increased with increasing postural challenge (Varghese et al., 2015).

**Table 4:** **Brain activity due to sensory challenges to static balance control**.

**Name, year**	**Balance challenge**	**Modality**	**Mobile**	**Spatial location**	**Activity**
Chang et al., 2016	Platform perturbations with and without synced VR in older adults	EEG	No	Parietal-occipital region	Gamma, beta bands
				Frontal-central region	Theta band
				Occipital lobe	Alpha band
Del Percio et al., 2007	Standing with eyes open or closed	EEG	No	Rt. ventral CP area	Alpha band ERD amplitude
Karim et al., 2013	Fixed floor, eyes open light/dark, sway-referenced floor, eyes open light/dark	fNIRS	No	Bi. Lat. temporal-parietal areas	Activation
Mitsutake et al., 2015	Head rotations on a rotating platform	fNIRS	No	Cz, T3, T4, F3, F4	Activation
Ouchi et al., 1999	Bipedal or unipedal stance; eyes open or closed	PET	No	Cerebellar anterior vermis, visual cortex, PFC	Activation
Petrofsky and Khowailed, 2014	Eyes open/closed, surface compliance, base of support in amputees vs. controls	EEG	No	Fz, F3, F4, Cz, C3, C4, POz, P3, and P4	Alpha, beta, and sigma band power
Pirini et al., 2011	Auditory feedback in eyes open vs. eyes closed scenarios	EEG	No	Rt inferior parietal	Alpha power
				Lt temporo-parietal, Lt temporo-occipital	Gamma power
Slobounov et al., 2015	Maintain balance in heel-to-toe stance while subjected to 2D or 3D VR moving room	EEG	No	Frontal midline	Theta power
Slobounov et al., 2013	Optical flow with various degrees of uncertainty	EEG	No	Frontal-central areas	Theta power
Tse et al., 2013	Eyes open/ closed; Firm/foam surface; Regular stance/heel-toe position	EEG	No	Parietal and central areas	Beta and Sigma band power
Varghese et al., 2015	Standing with eyes closed and feet together or feet heel-to-toe position	EEG	No	Cz	Amplitude of N100 evoked prior to balance reaction

*TM, treadmill; OG, overground; VR, virtual reality; lt., left; rt., right; bi. lat., bilateral; ERD, event related desynchronization*.

Chang et al. (2016) utilized frequency analysis and observed a modulation of alpha, beta, gamma, and theta band power in response to balance challenge in a virtual reality environment, particularly an increase in alpha power in occipital lobe in the virtual reality condition in both groups. Del Percio et al. (2007) also observed and larger amplitude of alpha band ERD in athletes in a closed-eye one and two leg balance task, compared to non-athletes. In contrast, Pirini et al. (2011) observed a decrease in alpha power during eyes-open task in the right inferior parietal area, which is in line with the increased attention required to perform a balance under degraded conditions. Petrofsky and Khowailed (2014) also observed an increase in activation with increased balance challenge, finding an overall signal power increased with a decrease in the amount of qality of sensory feedback. Likewise, Tse et al. (2013) observed an increase in beta and sigma bands in parietal and central areas with a more difficult balance challenge.

Using fNIRS, Karim et al. (2013) found increased activation of the bilateral temporal-parietal areas when both vision and perceptual information were degraded (eyes closed, swaying floors). Mitsutake et al. (2015) aimed to induce instability by requiring subjects to rotate their heads while on a rotating platform. Using fNIRS, they found that activation at the central, frontal and temporal cites were not significantly different during high or low speed rotations despite these differences in stability. This may indicate different types of instability may differentially affect cortical or spinal control mechanisms.

### Studies selected for RQ2

Initial searches yielded 47 studies investigating brain activity during both steady-state walking and dynamic balance control tasks. Given that brain activity during steady-state walking has been thoroughly reviewed by Hamacher et al. (2015), this review only includes the remaining 32 studies investigating brain activity under challenges to dynamic balance control. The balance challenge paradigms include mechanical perturbations, the use of cognitive dual-tasks, and the experimental manipulation of sensory inputs. Results can be found in Tables 5–**7**, respectively.

**Table 5:** **Brain activity due to mechanical challenges to dynamic balance control**.

**Name, year**	**Balance challenge**	**TM or OG**	**Modality**	**Mobile**	**Spatial information**	**Brain activity**
Beurskens et al., 2016	ST vs. DT: Motor or cognitive interference	TM PWS	EEG	Yes	FCz	Alpha band activity decreased during motor DT vs. ST
					FPz, Fz	Beta increased during motor vs. cognitive DT
Bradford et al., 2015	TM walking at specified levels of incline	TM Fixed	EEG	No	Sensorimotor, posterior parietal, ACC clusters	Higher theta power fluctuations across gait cycle in inclined walking conditions
					Lt. sensorimotor, ACC clusters	Greater gamma power during level walking
					Lt. and rt. sensorimotor cluster	Distinct alpha and beta fluctuations dependent on gait cycle for both walking conditions
Bruijn et al., 2015	Laterally stabilized while TM walking	TM Fixed	EEG	No	Bilateral premotor cortices	Higher beta power during stabilized walking in left premotor area specifically around push-off
Bulea et al., 2015	Steady state walking using an active or a passive TM	TM: Fixed vs. feedback driven	EEG	Yes	PFC and posterior parietal cortex	Low gamma band power increased during double support and early swing phases in active TM
					Sensorimotor cortex	Mu and beta band desynchronization during walking cycle
Clark et al., 2014b	Carrying tray, obstacles, and weighted vest tasks while walking in older adults	OG	fNIRS	No	PFC	Increased activation in walking phase
Haefeli et al., 2011	Obstacle navigation in dim lighting with audio cue to signal upcoming obstacle	TM Fixed	EEG	No	Oribital gyrus (BA 11) and medial frontal gyrus (BA 10)	Activation in preparation phase prior to stepping over obstacle
					Superior frontal gyrus (BA 9)	Activation in performance phase
Jaeger et al., 2016	External load applied during stepping movements	Stepping	fMRI	No	SMA-proper (BA4a), superior occipital gyrus (BA 18)	Activation in 0 load condition
					Vermis, S1/M1 (left BA 6), Thalamus	Activation in 20 load condition
					Insula, vermis, middle occipital gyrus, precuneus S2, thalamus, sup occ. gyrus	Activation in 40 load condition
Kurz et al., 2012	Forward vs. backward walking on TM	TM Fixed	fNIRS	No	SMA, pre-central gyrus, sup. parietal lobule	Increased activation in backward walking
					Pre-central gyrus and SMA	Maximal activation correlated with stride-time intervals in forward walking
Lin and Lin, 2016	Overground walking with wide, narrow, or obstacle path with and without n-back task	OG	fNIRS	No	PFC	Increased activation at beginning of task
Lu et al., 2015	Please refer to Table 6					
Maidan et al., 2015	Walking patterns known to cause FoG in PD patients with FoG and healthy controls	OG	fNIRS	No	Frontal activation (BA 10)	Decreased activation during turns without FoG episode in PD group
						Increased activation during anticipated turns before and during FoG episode
						No changes in activation in controls
Presacco et al., 2011	Real time visual feedback of lower limbs provided in order to avoid stepping on diagonal stripe on TM belt	TM PWS	EEG	No	Full scalp analysis	Higher delta, theta, and low beta spectral power during walking vs. rest
					Prefrontal, central, posterior-occipital, right, and left hemisphere regions of interest	Fluctuations in amplitude in EEG signals in low delta frequency band can predict gait kinematics
Presacco et al., 2012	Real time visual feedback of lower limbs provided in order to avoid stepping on diagonal stripe on TM belt	TM PWS	EEG	No	Pre-frontal, motor, parietal, and occipital areas	Standardized voltage level fluctuations over time can predict gait kinematics
Sipp et al., 2013	Heel-to-toe walking on a TM-mounted balance beam	TM Fixed	EEG	No	ACC, anterior parietal, superior DLPFC, medial sensorimotor cortex	Larger mean theta power during walking on balance beam vs. TM
					Lt. and rt. sensorimotor cortex clusters	Lower beta power during walking on balance beam vs. TM
					Lt. sensorimotor cortex	Visible indication on spectrogram when falling off beam
Varghese et al., 2016	APA for lateral weight shift or stepping task with/without preloading weight to the stance leg	Stepping	EEG	No	Mid fronto-central electrodes	Increase in amplitude of movement related potentials prior to initiation of postural adjustment
						Movement related potentials associated with APA onset
						ERD of mu and beta bands associated with APA onset

*TM, treadmill; OG, overground; PW, preferred walking speed; ST, single task; DT, dual task; APA, anticipatory postural adjustment; PD, Parkinson's Disease; FoG, freezing of gait; BA, Brodmann Area; lt., left; rt., right; bi. lat., bilateral; PFC, prefrontal cortex; DLPFC, dorsolateral prefrontal cortex; ACC, anterior cingulate cortex; SMA, supplementary motor area; ERD, event related desynchronization*.

#### Mechanical challenges

A total of 15 studies investigated the brain activity response due to mechanical challenges to dynamic balance control (See Table 5). Five studies utilized fNIRS paradigms to investigate frontal and prefrontal cortex activity. Clark et al. (2014b) used multiple mechanical challenges to increase the complexity of walking, including negotiating obstacles, carrying a tray and walking with a weighted vest. Prefrontal cortex (PFC) activation increased prior to walking for all three challenges compared to steady state walking. Increased prefrontal cortex activation during the weighted vest and obstacle conditions was also observed. Lu et al. (2015) also had subjects carry an object while walking and observed an increase in prefrontal cortex activation during task initiation, an increase in supplementary motor area (SMA) and premotor cortex (PMC) activation during the task. In addition, an increase in premotor cortex and supplementary motor area activation correlated with decreased gait performance.

Using a truly mobile fNIRS hardware system contained in a backpack, subjects walked over narrow, wide, or obstacle path conditions (Lin and Lin, 2016). At the beginning of each trial, regardless of path condition, average prefrontal cortex activation was higher and had larger variability compared to the end of the trial. Lastly, in healthy controls and Parkinson's Disease patients with freezing of gait (FoG), Maidan et al. (2015) monitored frontal activation during walking patterns known to cause FoG episodes. In the Parkinson's patients, frontal activation was decreased during turns without FoG episodes but increased during anticipated turns before and during FoG episodes. In the healthy controls, there was no change in frontal activation during turns.

Eight studies utilized EEG paradigms to investigate mechanical challenges to dynamic balance control. Bradford et al. (2015) required subjects to walk on a treadmill at specified inclines and observed theta power fluctuations that increased at steeper inclines in the ACC, sensorimotor, and posterior parietal clusters. Similarly, Sipp et al. (2013) found that heel-to-toe walking on a TM-mounted balance beam caused an increase in theta power in the ACC, anterior parietal, superior dorsolateral prefrontal cortex, and medial sensorimotor cortex. Lastly, Presacco et al. (2011) observed higher grand average spectral power within the delta and theta bands while subjects performed precision walking to avoid an obstacle on the treadmill belt. Beurskens et al. (2016) observed an alpha band decrease at the FCz electrode during dual-task condition requiring subject to walk and perform a motor interference task (preventing sticks in both hands from touching) compared to steady state walking. They also observed an increase in beta power in this motor-interference DT condition in the FPz and Fz electrodes. Bruijn et al. (2015) also observed an increase in beta power during stabilized walking in the premotor area specifically around push-off. However, Sipp et al. (2013) found a lower beta band power during balance beam walking in the sensorimotor cortex clusters. In general, Sipp et al. (2013) found an increase normalized time-frequency spectrogram in the ACC, parietal cortex, and dorsolateral prefrontal cortex time locked to gait cycle during the loss of balance and visible electrocortical indications on the spectrogram for the left sensorimotor cortex prior to a fall. Haefeli et al. (2011) had subjects avoid obstacles while walking under reduced vision conditions, with an audio cue to alert the subject of the obstacle, observing an increase in prefrontal cortex activity compared to steady-state walking. More specifically, prior to stepping over the obstacle, the EEG signal amplitude was enhanced in the orbital gyrus and medial frontal gyrus, the area of the brain responsible for processing of environmental stimuli. Also, during the performance phase (stepping over the obstacle) enhanced EEG signal amplitude was observed in the superior frontal gyrus, the brain area for monitoring motor performance. Presacco et al. (2012) also observed differential brain activity during different phases of the walking cycle. Utilizing EEG signal captured while subjects walked on a treadmill using visual feedback to avoid stepping on a white line on the treadmill belt, the authors found that the spectral power and signal lag served as inputs into a model that allowed for successful prediction of gait kinematics. Varghese et al. (2016) investigated the predictive nature of dynamic balance control by instructing subjects to laterally shift their weight and laterally step with and without preloading their non-stepping leg. The movement related potential (MRP) due to the lateral weight shift, the anticipatory postural adjustment (APA), was studied by giving the subjects an auditory cue to take a lateral step. Varghese et al. (2016) found a MRP and ERD of mu and beta bands prior to both the APA and the onset of foot-off during stepping in the fronto-central cortical areas. However, prior to the APA and during lateral weight shift, there was no difference in brain activity, suggesting cortical activity involved in predictive balance control is independent of context (weight shift vs. lateral step).

One study utilized an fMRI paradigm, applying various external loads during stepping movements in an fMRI to simulate ground reaction forces experienced during real walking (Jaeger et al., 2016). In the zero unloaded condition, activation was observed in the supplementary motor area, superior occipital gyrus. When 20% of the subject's body weight was applied, primary somatosensory cortex (S1)/Primary motor cortex (M1), vermis, and thalamus activation was observed. Lastly, at a higher load condition (40% of body weight), activation was seen in the insula, vermis, middle occipital gyrus, precuneus secondary somatosensory cortex (S2), thalamus, superior occipital gyrus.

#### Cognitive challenges

Twenty studies used cognitive dual-task paradigms to challenge dynamic balance control (See Table 6). Shine et al. (2013a,b) used fMRI-compatible steppers and a virtual reality hallway to evaluate walking in Parkinson's disease patients with and without FoG. Shine et al. (2013a) found activation of the cognitive control network (bilateral posterior parietal cortices, midline pre-supplementary motor area, bilateral anterior insula, medial temporal lobes, extra-striate visual cortex) in Parkinson's disease patients with and without FoG while walking. However, when cognitive load was modulated by a Stroop task, the FoG group had lower activation of the anterior insula, ventral striatum, pre-supplementary motor area, and subthalamic nucleus in FoG group compared to non-FoG group.

**Table 6:** **Brain activity due to cognitive challenges to dynamic balance control**.

**Name, year**	**Balance challenge**	**TM or OG**	**Modality**	**Mobile**	**Spatial information**	**Brain activity**
Al-Yahya et al., 2016	ST vs. DT (counting) walking in adults with chronic stroke	TM PWS	fNIRS	No	PFC	Increased activation in DT for both groups
Beurskens et al., 2014	Walking with visual or verbal memory task in young and elderly adults	TM PWS	fNIRS	No	PFC	Decreased activation in DT (visual) in elderly group
					PFC	Little change in PFC activation in DT in young group
Beurskens et al., 2016	ST vs. DT: motor or cognitive interference	TM PWS	EEG	Yes	Cz	Decreased alpha activity during cognitive DT
					FCz, Cz	Decreased beta activity decreased during cognitive DT
Clark et al., 2014b	Verbal task while walking in older adults	OG	fNIRS	No	PFC	Increased activation during walking phase
De Sanctis et al., 2014	Evaluate walking load on response inhibition with Go/No-Go Task	TM Fixed	EEG	No	O1/Oz/O2	Increase in P200 amplitude between sitting and walking
					FCz, Cz, CPz	Reduction in N200 amplitude during walking vs. sitting
					CPz	P300 amplitude reduced for walking
					FCz, Cz	P300 increased amplitude, reduced latency at higher walking speed
Doi et al., 2013	DT walking using verbal letter fluency task in older adults	OG	fNIRS	No	PFC	Increased activation during DT walking
Holtzer et al., 2011	WWT DT in young and old adults	OG	fNIRS	No	PFC	Increased activation in WWT compared with ST walking
						Greater activation in young vs. old group in DT condition
Holtzer et al., 2015	WWT DT in older adults	OG	fNIRS	No	PFC	Increase in activation during WWT condition
Holtzer et al., 2016	WWT DT in adults with and without neurological gait abnormalities	OG	fNIRS	No	PFC	Increased activation during WWT
Huppert et al., 2013	Lateral stepping based on congruent or incongruent information	Stepping	fNIRS	No	BA 46, BA 6, BA 4	Increased activation in incongruent trials
Kline et al., 2014	Brooks spatial WM task at multiple speeds	TM Fixed	EEG	No	Somatosensory association cortex	Alpha power increased prior to stimulus presentation
						Alpha power decreased during memory encoding
					Rt. superior parietal lobule and posterior cingulate cortex	Theta power decreased around memory encoding
Lau et al., 2014	Respond to target while sitting or walking on TM, with or without cognitive DT	TM Fixed	EEG	No	Sensorimotor Cortex	Effective connectivity weaker for walking than standing regardless of cognitive task
					PFC, posterior parietal cortex, ACC	Connectivity stronger for walking than standing only in cognitive DT condition
Lin and Lin, 2016	Please refer to Table 5					
Lu et al., 2015	Walking with motor task (carry water on tray) or cognitive task (subtraction)	OG	fNIRS	Yes	Left PFC	Increase in activation during preparation of DT conditions, maintained activation during cognitive task.
					SMA and PMC	Increased activation during both DT conditions
					PMC and SMA	Increased activation correlated with declines in gait performance
Malcolm et al., 2015	Go/No-go task while sitting or walking in young and old healthy adults	TM Fixed	EEG	No	Cz, FCz, and CPz	Decreased N200 amplitude for DT condition in young adults
						Reduced N200 latency for DT condition compared in young adults
						Reduced P300 latency compared to sitting condition
Mirelman et al., 2014	Walking while counting forward, walking with serial 7's	OG	fNIRS	No	Fp1 and Fp2	Increased activation with increased task difficulty.
Osofundiya et al., 2016	DT walking (WWT), simple walking, and precision walking in older adults (obese and controls)—Holtzer 2011, Verghese 2002	OG	fNIRS	No	PFC	Oxygenation levels were higher in complex ambulatory tasks
						Higher oxygenation levels in obese group (performance metrics were the same)
Shine et al., 2013a	Stop-signal task in a VR environment to navigate a corridor using foot pedals. Cognitive load modulated by Stroop task. Older adults with PD, with or without FoG	Stepping	fMRI	No	Bi. lat. posterior parietal cortices, midline pre-SMA, bi. lat. anterior insula, medial temporal lobes, extra-striate visual cortex	Activation in both groups when walking with VR paradigm
					Bi. lat. anterior insula, ventral striatum, pre-SMA, lt. subthalamic nucleus	Lower activation during cognitive load condition while stepping in FoG group
Shine et al., 2013b	Stop-signal task in a VR environment to navigate a corridor using foot pedals. Cognitive load modulated by Stroop task. Older adults with PD, with or without FoG	Stepping	fMRI	No	Lft CCN and ventral attention network	Activation in both groups during task performance
					Bilateral cognitive control network	Increased connectivity in both groups during task performance
					Motor network	Activation during high cognitive load condition, to lesser extent in FoG group
					Motor network and left CCN	Increased connectivity during high cognitive load
					Basal ganglia network and CCN in each hemisphere	Decoupling in FoG group, associated with freezing event
Takeuchi et al., 2016	Walking while playing on smart phone	OG	fNIRS	Yes	PFC	No difference in activation during smartphone use while walking between young and old groups
						Differential activation in old and young groups correlated to walking acceleration, step time, and game mistakes

*TM, treadmill; OG, overground; PWS, preferred walking speed; ST, single task; DT, dual task; WWT, walking while talking; WM, working memory; VR, virtual reality; PD, Parkinson's Disease; FoG, freezing of gait; BA, Brodmann Area; lt., left; rt., right; bi. lat., bilateral; PFC, prefrontal cortex; ACC, anterior cingulate cortex; SMA, supplementary motor area; CCN, cognitive control network*.

Shine et al. (2013b) found that Parkinson's patients, both with and without FoG, used left cognitive control network (CCN) (left prefrontal cortex, dorsolateral prefrontal cortex, ventrolateral prefrontal cortex) and ventral attention network (anterior insula, dorsal cingulate) and exhibited increased connectivity between the bilateral cognitive control networks. There was increased connectivity of the motor network (pre and post-central gyrus, right supplementary motor area) and left CCN when walking with high cognitive load. Lastly, there was decoupling of the basal ganglia network (caudate, rostral cingulate) and CCN in each hemisphere in the FoG group which were associated with the occurrence of motor arrests, indicating impaired communication between these networks in FoG. Shine et al. (2013a,b) observed activation of brain regions in the locomotor pathways. However, they also observed activation and connectivity with attention and cognitive control networks required to perform cognitive tasks. Additionally, impaired connectivity seen in FoG provides evidence toward the use of compensation mechanisms and/or additional brain regions in walking in those with neurological disorders.

Thirteen studies utilized fNIRS to investigate cognitive challenges during dynamic balance control, focusing on activation of the prefrontal cortex, supplementary motor area, and premotor cortex. Holtzer et al. (2011) and Holtzer et al. (2015) found increased activation of the prefrontal cortex during a walking while talking task compared to normal walking conditions in older adults. Holtzer et al. (2016) used a similar task paradigm in healthy controls and patients with neurological gait abnormalities, finding that increased prefrontal cortex activation in healthy controls was correlated with high cognitive performance and slow gait speed. In addition, increased prefrontal cortex activation in the group with neurological gait abnormality correlated with low cognitive performance and fast gait speed. Osofundiya et al. (2016) observed higher prefrontal cortex oxygenation levels during complex ambulatory tasks (walking while talking and precision stepping). Doi et al. (2013) also found increased prefrontal cortex activation during a verbal fluency task while walking compared to single task (ST) condition. Using a verbal fluency task, Clark et al. (2014b) found an increase in prefrontal cortex activation compared to single-task walking. In a walking while counting dual-task condition, Al-Yahya et al. (2016) found increased activation of the prefrontal cortex compared to single task walking in older adults with chronic stroke. Lu et al. (2015) found increased activation of the left prefrontal cortex, supplementary motor area, and premotor cortex during walking while subtracting conditions and that increased premotor cortex and supplementary motor area activation were correlated with declines in gait performance. Similarly, increased difficulty of a counting dual-task increased anterior prefrontal cortex activity (Mirelman et al., 2014). Beurskens et al. (2014) used visual and verbal memory demand dual-task conditions while monitoring prefrontal cortex activation in elderly and young subjects. Similar to Holtzer et al. (2011) and Doi et al. (2013), the dual-task condition led to an increase in prefrontal cortex activation in the elderly subjects. However, in young adults, there was a smaller increase in prefrontal cortex activation from single task to dual-task conditions.

More complicated paradigms have also been used to investigate brain activity in walking or stepping. Huppert et al. (2013) required subjects to step left or right according to incongruent information (conflicting location and direction of arrow on screen) or congruent information (location and arrow matched), and found increased activation in dorsolateral prefrontal cortex, premotor cortex, supplemental motor area, and precentral gyrus in incongruent trials, which require more attentional control. Lin and Lin (2016) had subjects walk over ground on wide, narrow, or obstacle pathways and with or without a cognitive load (n-back task), and found a decrease in prefrontal cortex activation at higher cognitive loads, similar to Beurskens et al. (2014) and Shimada et al. (2013). Lastly, Takeuchi et al. (2016) observed no difference in prefrontal cortex activation between young and old groups while they played a game on their smartphone while walking. However, differential activation patterns in the right, left, and middle prefrontal cortex were observed to be correlated with gait speed, game mistakes, and step timing in the old and young groups.

Although these fNIRS studies investigate a range of cognitive dual-task conditions using both over ground and treadmill balance paradigms, there is no agreement on the activation of prefrontal cortex, supplementary motor area, and premotor cortex during dual-task conditions. Some studies found an increase in activation in dual-task conditions (Holtzer et al., 2011; Doi et al., 2013; Clark et al., 2014b; Al-Yahya et al., 2016), while others found a decrease (Shimada et al., 2013; Beurskens et al., 2014; Lin and Lin, 2016). In addition, although aging negatively affects balance control, Takeuchi et al. (2016) did not find a difference in prefrontal cortex activation between young and older adults while playing a game and walking.

Seven studies utilized EEG paradigms to investigate the impact of cognitive challenges on dynamic balance control. In a dual-task condition with a cognitive interference task, Beurskens et al. (2016) found a decrease in alpha band power over the Cz electrode and beta activity over the FCz and Cz electrodes compared to normal walking. However, beta band power increased during the cognitive dual-task over the FPz and Fz electrodes. Kline et al. (2014) had young healthy subjects perform a Brooks spatial working memory (WM) task at different treadmill speeds and found that there was an increase in alpha power prior to stimulus presentation followed by a decreased in alpha power during memory coding in the somatosensory association cortex. A decrease in theta power around memory encoding in the right superior parietal lobule and posterior cingulate cortex was also observed. Additionally, BA 7, BA 31, BA 5, and 6 exhibited power fluctuations time-locked to memory encoding during cognitive tasks. However, no distinct changes in brain activity or working memory task performance were observed due to different walking speeds. De Sanctis et al. (2014) evaluated the impact of walking on a go/no-go task that requires response inhibition in young adults, finding an increase in P200 amplitude in walking conditions (occipital lobe). A reduced N200 amplitude (at FCz, Cz, and CPz) and P300 amplitude (at CPz) was observed on trials requiring inhibition while walking as compared to sitting. Increased P300 amplitude and decreased latency was observed in faster walking conditions (at FCz). These differences in ERPs were not accompanied by performance decrements in dual-task conditions, suggesting the use of compensation mechanisms to appropriately allocate attentional resources to perform both tasks (De Sanctis et al., 2014). Malcolm et al. (2015) also used a go/no-go task in young and older adults while walking or sitting, finding a decreased N200 amplitude for dual-task condition and earlier P300 latency over the Cz, FCz, and CPz electrodes in young adults, similar to De Sanctis et al. (2014). Young adults exhibited a decreased N200 latency, while older adults showed an increase in P300 amplitude, indicating that older adults may have less flexibility of resource allocation in multi-task conditions (De Sanctis et al., 2014). Lau et al. (2014) investigated effective connectivity of the sensorimotor cortex and non-sensorimotor areas of the brain while subjects stood or walked on a treadmill with or without a cognitive challenge. They found that the effective connectivity of sensorimotor cortex was weaker for walking than standing, regardless of dual-task condition, which may point to a more automatic nature of walking execution. However, effective connectivity of non-sensorimotor areas (prefrontal cortex, posterior parietal cortex, and ACC) was stronger in the cognitive dual-task condition for walking than standing.

#### Sensory challenges

The three studies investigating sensory challenges to balance control all observed modulation in prefrontal cortex activity (Haefeli et al., 2011; Clark et al., 2014a,b) (See Table 7). In a dimly lit walking environment, there was an increase in prefrontal cortex activity during preparation and performance of the walking task compared to a regularly lit condition (Clark et al., 2014b). While it has been shown that steady-state walking in older populations is less automatic and increases the need for attentional control and cognitive processes compared to younger populations, Clark et al. (2014a) had older adults with mild mobility deficits walk and mild somatosensory deficits walk on a treadmill and over ground while wearing normal shoes, and under enhanced somatosensory conditions wearing textured insoles, and with no shoes. There was a reduction in prefrontal cortex activity when subjects wore textured insoles compared to normal shoes for both treamill and overground walking. Also, walking with no shoes reduced prefrontal cortex activity for treadmill walking only compared to walking with normal shoes. Enhancing somatosensory information led to a reduction in prefrontal activation compared to control conditions, suggesting a more automatic process is being used perform the walking task. In contrast, under degraded visual conditions, Clark et al. (2014b) and Haefeli et al. (2011) observed increased prefrontal cortex activity.

**Table 7:** **Brain activity due to sensory challenges to dynamic balance control**.

**Name, year**	**Balance challenge**	**TM or OG**	**Modality**	**Mobile**	**Spatial information**	**Brain activity**
Clark et al., 2014a	Sensory enhancement (textured insoles or bare feet) in older adults	TM vs. OG	fNIRS	No	Bilateral PFC	Reduction in activation for textured insole and OG conditions
						Decrease in activity in no shoe walking
Clark et al., 2014b	Dim lighting while walking in older adults	OG	fNIRS	No	PFC	Increased activation during preparation and performance phase
Haefeli et al., 2011	Please refer to Table 5					

*TM, treadmill; OG, overground; PFC, prefrontal cortex*.

### Studies selected for RQ3

#### Movement artifact identification and removal

Eight EEG studies focused on the identification and/or removal of movement artifact from the brain signal collected while walking (see Table 8). Gwin et al. (2010) used a visual oddball paradigm while subjects walked or ran on a treadmill to elicit an ERP. In the walking condition, the ERP was nearly identical before and after movement artifact removal. However, in the running condition, the ERP was only identifiable after using a template regression artifact removal process. Gramann et al. (2010) also investigated the feasibility of using more mobile EEG hardware (a single tether connected electrodes to equipment) while subjects were walking or standing. The authors were able to identify the P300 and N100 ERP components evoked by a visual oddball paradigm over the Cz, Fz, and Pz electrodes. Oliveira et al. (2016) aimed to establish a protocol to validate the efficacy of EEG hardware and software systems in mobile neuroimaging using an auditory oddball paradigm in sitting and walking conditions. The authors recommended using epoch rejection rate, pre-stimulus noise, signal-to-noise ratio, and amplitude variance across the P300 event window to evaluate EEG hardware systems and artifact identification and removal efficacy in walking studies. In a different approach, Lau et al. (2012) used a weighted phase lag index across all channels and recovered a P300 response to a target stimulus while subjects stood or walked on a treadmill.

**Table 8:** **Neuroimaging studies investigating feasibility EEG signal acquisition during dynamic balance control tasks**.

**Name, year**	**Balance challenge**	**TM or OG**	**Modality**	**Mobile**	**Spatial information**	**Analysis**
Gramann et al., 2010	Visual oddball response task while standing/walking	TM Fixed	EEG	No (tether)	Fz, Cz, Pz	Identification of P300 and N100 amplitudes due to visual oddball stimulus
Gwin et al., 2010	Visual oddball task while walking or running	TM Fixed	EEG	No	Mediofrontal clusters	Identification of gait-related artifact in ERP during running
Oliveira et al., 2016	Auditory oddball task seated and walking	TM Fixed	EEG	Yes	Fpz, F3, Fz, F4, C3, Cz, C4, P3, Pz, P4, O1, O2	Epoch rejection rate, pre-stimulus noise, signal-to-noise ratio, P300 amplitude
Lau et al., 2012	Response to target stimulus while walking	TM Fixed	EEG	No	Global	Identification of P300 response in walking condition
Castermans et al., 2014	Barefoot walking	TM Fixed	EEG	No	Cz, Oz, T8	Harmonics in accelerometer and EEG signals (delta, theta, alpha bands)
Snyder et al., 2015	Walking at set speeds with silicone cap	TM Fixed	EEG	No	Global	Movement artifact remains in EEG signal following ICA and dipole fitting
Kline et al., 2015	Walking at set speeds with silicone cap	TM Fixed	EEG	Yes	E12, A19, G11, C19, A1	Movement artifact varies with speed, subject and electrode location
Nathan and Contreras-Vidal, 2016	Walking at set speed	TM Fixed	EEG	Yes	Cz, Oz, T8	No large amplitude spikes in spectral signals corresponding stepping frequency (accelerometer signal)
						Strong wavelet coherence between delta band and accelerometer for higher walking speeds.

*TM, treadmill; ERP, event related potential; ICA, Independent Component Analysis*.

Four studies have focused on characterizing the walking related movement artifact. Castermans et al. (2014) used barefoot walking on a treadmill while collecting both EEG and accelerometer signals. The accelerometer was mounted on the subject's head and showed increased activity corresponding to walking events (heel-strike and double support phase). Additionally, analysis of the EEG and accelerometer signals showed harmonics up to 15 Hz in the EEG signal, with the potential to impact the delta, theta and alpha frequency bands. Furthermore, the signal correlation varied for each of the three EEG electrode locations (Cz, Oz, T8), suggesting that a uniform filtering method could not be used across all scalp electrodes (Castermans et al., 2014). Kline et al. (2015) and Snyder et al. (2015) used a silicone swim cap to block all electrocortical activity, thus measuring only movement artifact while subjects walked on a treadmill at different speeds. Like Castermans et al. (2014) and Kline et al. (2015) collected accelerometer data, however, there was poor correlation between the EEG and accelerometer signals. Furthermore, there was variation in movement artifact at different walking speeds, electrode locations, and in each subject. Snyder et al. (2015) used independent component analysis (ICA) and dipole fitting to localize movement artifact. ICA is an advanced mathematical tool that separates a signal into statistically independent components, potentially separating the EEG signal into independent cerebral sources and artifact sources (Makeig et al., 1996). Dipole fitting of the pure movement artifact signal accurately localized 99% of the independent components (ICs) originating outside the brain. The remaining 1% of ICs remained in the signal and had similarities with neural sources, revealing opportunities to completely remove movement artifact during walking. In contrast to the focus of Kline et al. (2015) and Snyder et al. (2015) on movement artifact only, Nathan and Contreras-Vidal (2016) analyzed the EEG signal and its relation to an accelerometer signal which was collected while subjects walked at various speeds. In this study, there were no large spectral amplitude spikes corresponding to the stepping frequency, which was obtained from the accelerometer signal. This finding refutes the analysis in Castermans et al. (2014). Wavelet coherence analysis revealed strong coherence between the EEG signal within the delta band and the accelerometer for higher walking speeds, which suggests the emergence of movement artifact within the EEG signal. However, after artifact subspace reconstruction, an automated artifact rejection process that uses baseline data and principal component analysis to remove transient and high amplitude artifact (i.e., muscle movements), this delta band coherence was not observed in the average subject data. This study suggests that movement artifact may not contaminate EEG data collected at slower walking speeds when using mobile neuroimaging equipment (Nathan and Contreras-Vidal, 2016).

#### Signal processing of fNIRS data

Table 9 summarizes the pre-processing and movement artifact removal methods of 20 studies using fNIRS for neuroimaging. Two studies used wavelet-minimum description length de-trending algorithms to remove global trends including artifacts due to respiration, heartbeat, and vasoconstriction. Five studies used principal (PCA) or independent (ICA) component analysis, or their combination, to remove environmental and equipment noise and signal drift. However, the majority of these studies did not use computational methods to remove artifacts from the acquired signal.

**Table 9:** **Signal processing and artifact removal methods for fNIRS studies**.

**Name, year**	**Pre-processing**	**Removal of movement artifact**
Al-Yahya et al., 2016	Low-pass filter at 0.67 Hz cutoff frequency	
Beurskens et al., 2014	Gaussian/Hemodynamic response function lowpass filter	Wavelet-minimum description length algorithm
Caliandro et al., 2015	Low pass filter (0.1 Hz)	
Doi et al., 2013	Low pass filter (0.5 Hz)	
Fujita et al., 2016	Low pass filter (0.5 Hz)	
	High pass filter (0.01 Hz)	
Holtzer et al., 2011	Low pass filter (FIR, 0.14 Hz)	ICA and PCA
Holtzer et al., 2015, 2016	Low pass filter (FIR, 0.14 Hz)	Inspection to remove signal artifact
Huppert et al., 2013	Low pass filter (0.8 Hz)	
	Series of discrete cosine transform terms	
Karim et al., 2013	Series of discrete cosine transform terms	
Kim et al., 2016		Gaussian smoothing. Wavelet minimum description length algorithm.
Koenraadt et al., 2014	Low pass filter (Butterworth, 1.25 Hz)	Short separation channels and scaling factor used to normalize data per individual
	High pass filter (Butterworth, 0.01 Hz)	
	Low pass filter (Butterworth, 1 Hz)	
Kurz et al., 2012	High pass filter (0.01 Hz)	PCA, removing components <0.25 correlation with reference waveform
Lin and Lin, 2016	Low pass filter (FIR, 0.2 Hz)	
Lu et al., 2015	Removal of noisy channels using coefficient of variation Bandpass filter (0.01–0.2 Hz)	PCA and Spike Rejection
Maidan et al., 2015	Low pass filter (FIR, 0.14 Hz)	
Mihara et al., 2008	High pass filter (0.05 Hz)	Gaussian function
Mihara et al., 2012	High pass filter (0.03 Hz)	
Mirelman et al., 2014	Low pass filter (FIR, 0.14 Hz)	
Takeuchi et al., 2016	Bandpass filter (0.01–0.5 Hz)	Rapid changes in oxyHb concentration were removed

*FIR, finite impulse response; ICA, independent component analysis; PCA, principle component analysis; SD, standard deviation*.

#### Signal processing of EEG data

Table 10 summarizes the pre-processing, spatial filtering, and movement artifact identification and removal methods used by the 54 EEG studies included in this review. For data pre-processing, all studies except three reported using high-pass, low-pass, or bandpass filters to exclude certain frequency ranges. Gwin et al. (2010) described criteria for removing noisy channels prior to data analysis: Standard deviation (*SD*) >1,000 μV, kurtosis >5 *SD* from mean, or poor correlation (*r* < 0.4) with neighboring channels for >1% of time samples. Ten additional studies used these criteria for removing noisy channels prior to further analysis. Twenty-five studies used spatial filtering techniques, including Laplacian estimation (3), referencing to a common average (21), and bi-lateral referencing (1).

**Table 10:** **Signal processing and artifact removal methods for EEG studies**.

**Name, year**	**Pre-processing**	**Spatial filtering**	**Movement artifact removal**
Adkin et al., 2008	Bandpass filter (0.1–10 kHz)		
	Low-pass filter (30 Hz cutoff)		
Adkin et al., 2006	Bandpass filter (0.0001–30 Hz)		Manually removed trials with artifact
Beurskens et al., 2016	Bandpass filter (0.5–45 Hz)	Reference using common reference	Visual inspection and semiautomatic artifact rejection (±100 μV)
Bradford et al., 2015	Noisy channels removed		AMICA and DIPFIT
Bruijn et al., 2015	High-pass filter (3 Hz Butterworth)	Average common reference	ICA
	Band-stop filter (50, 100, 150, and 250 Hz)		
Bulea et al., 2014	High pass filter (Butterworth, 0.05 Hz)	Common average reference	Artifact subspace reconstruction (uses PCA to clean data)
	Bandpass filter (Butterworthy, 0.1–4 Hz)		
Bulea et al., 2015	High pass filter at 1 Hz	Re-referenced to common average of the remaining channels	Artifact subspace reconstruction (uses PCA to clean data). AMICA and DIPFIT
	Power line noise remove		
	Noisy channels removed		
Chang et al., 2016	Bandpass filter (70 Hz–DC)		
	Notch filter (60 Hz)		
	Bandpass filter at (0.1–50 Hz)		
De Sanctis et al., 2014	Bandpass filter (0.05–100 Hz)	Re-referenced offline to an average reference	Automatic artifact rejection (±100 μV)
	Bandpass filter (0.5–30 Hz)		
Del Percio et al., 2007, 2009	Bandpass filter (0.01–100 Hz)	Laplacian estimation	Autoregressive method to remove ocular artifact
Gramann et al., 2010	High pass filter (1 Hz)	Re-referenced offline to an average reference	ICA, AMICA and DIPFIT
	Noisy channels removed		
Gwin et al., 2010	High pass filter (1 Hz)	Re-referenced offline to an average reference	Moving average method
	Noisy channels removed		
Gwin et al., 2011	High pass filter (1 Hz)	Re-referenced offline to an average reference	Rejected time periods with substantial artifact. AMICA and DIPFIT
	Noisy channels removed		
Haefeli et al., 2011	Bandpass filter (1–30 Hz)	Average reference as recording reference	All artifacts exceeding ± 80 μV excluded, then ICA
Huang et al., 2014	AC amplifier with cutoff freq 5–450 Hz		Visually inspected and removed artifacts. PCA prior to ERP spectral analysis
	Bandpass filter (100 Hz)		
	Low pass filter (40 Hz)		
Hülsdünker et al., 2015, 2016	Band pass filter (2–120 Hz)	Re-referenced to a common average reference.	Semiautomatic rejection algorithm followed by ICA
Jacobs et al., 2008	Bandpass filter (0.05–60 Hz)		
Kline et al., 2014	High pass filter (1 Hz)	Re-referenced the remaining channels to an average reference	AMICA and DIPFIT
	Noisy channels removed		
Kline et al., 2015	High pass filter (1 Hz)	Re-referenced the remaining channels to an average reference.	Moving average method
			Wavelets
	Rejected epochs > 3 standard deviations from the means of the gait event times		Moving Average and Wavelets
		EEG movement artifact compared to accelerometer signal	
Lau et al., 2014	Highpass filter (1 Hz).	Re-referenced the remaining channels to an average reference	Moving average method
	Filter line noise (60 Hz)		
	Noisy channels removed		
Lau et al., 2012	Highpass filter (1 Hz).	Channels were then re-referenced to an average of the remaining channels.	Weighted Phase Lag Index
	Filter around 4+/−2H		
	Noisy channels removed		
Little and Woollacott, 2015	Notch filter (60 Hz)		Artifact detection algorithm and visual inspection
	High pass filter (30 Hz)		
	Low pass filter (0.1 Hz)		
Luu et al., 2016	Adaptive filter		Artifact subspace reconstruction (uses PCA to clean data)
Malcolm et al., 2015	Low pass filter (Butterworth 7 Hz); Bandpass filter (1–30 Hz)	Re-referenced to an average reference	Artifact rejection criterion (±75 μV)
Marlin et al., 2014	Filter (DC–300 Hz)		Visual inspection to remove ocular artifact. PCA and ICA
	Low pass filter (30 Hz)		
Mierau et al., 2015	Butterworth IIR filter,Bandpass filter (2–30 Hz)	Laplacian interpolation	Corrected for ocular artifacts
Mochizuki et al., 2008, 2009a,b	Low pass (200 Hz)		Removal of ocular artifact
	High pass (DC)		
	Low pass (30 Hz)		
Nathan and Contreras-Vidal, 2016	Low pass (DC–1,000 Hz)		Artifact subspace reconstruction (uses PCA to clean data)
Oliveira et al., 2016	High pass filter (1 Hz)		Removed frame sequences with large artifacts due to lost packets during wireless telemetry and EMG. ICA
	Notch filter using Cleanline		
Petersen et al., 2012	Filter (1–500 Hz)	Re-referenced to a common average reference	ICA
	Removed significant drift or >50 Hz noise		
Petrofsky and Khowailed, 2014	Bandpass filter (0.1–65Hz)		Mean wavelet coefficients per frequency bin. Linear discriminant function analysis to classify eye blinks
	Notch filter (60z)		
	Removed amplitude saturation		
Pirini et al., 2011			Manual cleaning and ICA
Presacco et al., 2011, 2012	Bandpass filter (0.1–100 Hz)		None
	Bandpass filter (Butterworth 0.1–2 Hz)		
Quant et al., 2004	Bandpass filter (1–10,000 Hz)		Visually inspected for artifact and averaged over each subject
Quant et al., 2005	Bandpass filter (1–10,000 Hz); Butterworth low-pass filter (30 Hz)		Visually inspected for artifact and averaged over each subject
Sipp et al., 2013	Bandpass filter (DC–104 Hz)	Remaining channels were average referenced.	Moving average method
	High pass filter (1 Hz)		
	Noisy channels removed		
Slobounov et al., 2015	Filter (2–30 Hz)		Manual check for artifacts and eye blinks
Slobounov et al., 2013	Filter (4–30 Hz)		Checked and corrected for artifacts and eye blinks removed
Slobounov et al., 2009	Filter (70 Hz)		ICA, Morlet wavelet transformation
Slobounov et al., 2008	Filter (100 Hz)		Visual inspection
Slobounov et al., 2005	Filter (100 Hz)		Ocular artifact reduction through NeuroScan software
Smith et al., 2012, 2014	Low pass filter (40 Hz)	Re-referenced to a common average reference	ICA
Snyder et al., 2015	Low pass filter (1 Hz)	Re-referenced to average	AMICA followed by DIPFIT
	Noisy channels removed		
Storzer et al., 2016	Bandpass filter (1–100 Hz)	Re-referenced against the common average	Visual inspection then ICA
	Bandstop (49–51 Hz)		
Tse et al., 2013	Bandpass filter (0.1–65 Hz)		Mean wavelet coefficients per frequency bin. Linear discriminant function analysis to classify eye blinks
	Notch filter (60 Hz)		
Varghese et al., 2016	Bandpass filter (0.05–50 Hz)		ICA
Varghese et al., 2015	Bandpass filter (DC–300 Hz)		ICA
	Bandpass filter (2–50 Hz)		
Varghese et al., 2014	Bandpass filter (1–30 Hz)		ICA

*ICA, Independent component analysis; AMICA, Adaptive mixture ICA; PCA, Principal component analysis; DIPFIT, dipole source localization of independent components; ERP, event related potential*.

The literature reports a multitude of techniques to identify and remove ocular, muscular, and/or movement artifact during dynamic balance tasks, ranging from visual inspection (10) to sophisticated computational techniques. Petrofsky and Khowailed (2014) and Tse et al. (2013) used logistic discrimination function analyses tuned using an EEG database from sleep-deprived adults to identify eye blink regions, which were subsequently replaced by mean wavelet coefficients from nearby non-contaminated regions (Berka et al., 2007). Three studies defined and rejected time periods having substantial artifact using a criterion (>0.8) on the z-transformed power across all channels (Gwin et al., 2011; Sipp et al., 2013; Lau et al., 2014). Gwin et al. (2011) presented a moving average artifact removal method for EEG data collected during walking. Here, time-warped strides were averaged before and after a specific foot strike event. Then this time-warped average stride data was subtracted from the new data for a current stride. This was also used in Kline et al. (2015) explored the combination of this moving average approach with a wavelet technique that removed signal content at frequencies below 8 Hz and applied the wavelets to the whole stride using Daubechies four wavelets.

However, approximately half the studies (i.e., a total of 23) used conventional ICA or adaptive mixture ICA (AMICA) to identify and remove artifacts from EEG data collected during walking. Hülsdünker et al. (2015) and Hülsdünker et al. (2016) identified and removed ocular or muscular artifacts based on the cortical mapping, frequency spectrum, and time course components using ICA. Bruijn et al. (2015) described the following criteria for categorizing and removing components not associated with brain activity: Muscle artifact (50–100 Hz mean power larger than that in the beta and/or alpha bands), eye-blink artifacts (median frequency <3 Hz and the topo map corresponded to eye components), and movement artifacts (frequency spectrum at the harmonics of stride frequency). After performing ICA or AMICA, several studies also used a source localization algorithm, DIPFIT, to refine artifact identification and removal. Gramann et al. (2010) evaluated each independent component for location within the head model and evaluated the residual variance between the scalp projection through a head model and scalp map. Gwin et al. (2011) used a similar approach but excluded components if the current dipole model to scalp accounted for less than 80% of the scalp variance - criteria used by six other studies (Sipp et al., 2013; Kline et al., 2014; Lau et al., 2014; Bradford et al., 2015; Bulea et al., 2015; Snyder et al., 2015). Lastly, 6 studies used PCA to identify and remove movement artifact (Bulea et al., 2014, 2015; Huang et al., 2014; Marlin et al., 2014; Luu et al., 2016; Nathan and Contreras-Vidal, 2016).

## General discussion and conclusion

This study reviewed 83 articles using neuroimaging modalities to investigate the neural correlates underlying static and dynamic human balance control, with aims to support future mobile neuroimaging research in the balance control domain. Images demonstrating the use of mobile neuroimaging modalities integrated with a dynamic balance control tasks can be found in Kline et al. (2015). Likewise, examples of study paradigms using non-mobile neuroimaging modalities within this domain can be found in Chang et al. (2016) and Gramann et al. (2010).

This review found that static balance control was largely examined with sensory or mechanical balance paradigms. There were relatively few static paradigms that utilized cognitive challenges to study the neural components of balance. Additionally, all but one static balance study using sensory challenges, such as using open and closed eye conditions, were performed on healthy, young adults. Analysis of brain activity during standing balance control included perturbation evoked ERPs and frequency analysis, with findings of increased activation during balance challenges, regardless of challenge type (sensory, cognitive, or mechanical). Activation of the prefrontal cortex (PFC), supplementary motor area (SMA), and Premotor cortex (PMC) frequently occurred in response to the static balance challenges. Although more common in dynamic balance control studies, seven of the static balance control studies invoking mechanical perturbations used advanced signal processing methodologies such as ICA to reduce movement artifacts (Mochizuki et al., 2009a; Smith et al., 2012, 2014; Marlin et al., 2014; Varghese et al., 2014, 2015; Hülsdünker et al., 2016).

In dynamic balance control paradigms, there were many cognitive dual-task conditions and very few sensory and mechanical balance challenges. The mechanical balance challenges included obstacles or challenge walking scenarios, such as using a balance beam. However, none of these studies used surface perturbations as seen in the static paradigms. Lastly, almost a third of the dynamic balance tasks included over ground walking instead of treadmill walking. Analysis of brain activity included activation of the prefrontal cortex, supplementary motor area, and SMC and frequency band analysis at different time points in gait cycle and task execution process. These areas of brain activation overlap with the direct and indirect locomotor pathways proposed by la Fougere et al. (2010). Given the dual-task nature of dynamic balance control challenges, it is not surprising that there is involvement of the prefrontal cortex, which is associated with the indirect locomotor pathway (la Fougere et al., 2010; Zwergal et al., 2013; Hamacher et al., 2015). Lastly, the majority of the EEG studies in this review used advanced signal processing methods to identify and remove movement artifact in the acquired brain signal, specifically by using ICA. Several studies have explicitly investigated and attempted to characterize the movement artifact due to steady-state walking and the feasibility of using these advanced signal processing methods for research in this domain.

### Mobile neuroimaging methods in static and dynamic balance control

Mobile neuroimaging was used in eight studies to investigate the brain activity associated with balance challenge tasks. Five studies used wireless EEG systems and dynamic balance control paradigms on treadmills (Bulea et al., 2015; Kline et al., 2015; Beurskens et al., 2016; Nathan and Contreras-Vidal, 2016; Oliveira et al., 2016), one study used wireless EEG in static balance control (Bulea et al., 2014), and two investigated dynamic balance control using paradigms pairing mobile fNIRS systems and over ground walking (Lu et al., 2015; Takeuchi et al., 2016). As most of these mobile neuroimaging studies paired walking with a cognitive or motor task, it is clear that the broad range of human balance control paradigms have not been fully investigated with mobile modalities. Given the innovative nature of this mobile technology, this is to be expected.

Interestingly, only two studies investigated brain activity in both static and dynamic balance control tasks (Lau et al., 2012; Mirelman et al., 2014). Neither of these studies used a mechanical perturbation to challenge balance. However, many static balance control paradigms use perturbations that invoke a feet-in-place response in which subjects use only postural sway strategies to maintain upright balance (Adkin et al., 2008). However, perturbations that exceed this feet-in-place threshold, and thereby elicit a stepping response, can potentially be used to develop a deeper understanding of the neural mechanisms involved in the transition between static and dynamic balance control. Two main recommendations for future mobile neuroimaging research include:

1. Researchers utilizing mobile EEG and/or fNIRS systems should focus on pairing these modalities with a broader range of dynamic balance control challenges, such as sensory perturbations. Additionally, the use of these mobile modalities in static balance control challenges is minimal, providing an opportunity for further investigation and direct comparison to findings from tethered neuroimaging systems.
2. Mobile neuroimaging modalities should be paired with balance control paradigms that go beyond a feet-in-place reaction (Slobounov et al., 2009; Varghese et al., 2016). Perturbations that require the subject to maintain balance control in an upright stance but require a stepping response may provide insights into mechanisms required in the transition between static and dynamic balance control.

### Multi-imaging paradigms in balance control domain

All neuroimaging methods have advantages and disadvantages. By concurrently using multiple imaging modalities within the same study, we could develop a deeper understanding of the temporal and spatial dynamics of brain activity in the balance control domain. Within this review, one study analyzed dynamic balance control through fNIRS and fMRI (Al-Yahya et al., 2016). In this study, real gait was paired with fNIRS methods while isolated ankle movements were paired with fMRI. The latter task excluded this condition from inclusion in this review. Three studies in this review implemented MRI-compatible steppers to allow for active leg movements that closely resemble gait (Shine et al., 2013a,b; Jaeger et al., 2016).

Additionally, both fMRI and fNIRS are indirect, hemodynamic imaging methods that have poor temporal resolution. In contrast, direct, electrocortical modalities such as EEG are known to have high temporal resolution. Within other research domains, such as neural mechanisms of emotion, both fMRI and EEG are used to capitalize on the advantages of both indirect and direct imaging methods. The challenge in the balance control domain is the development of research paradigms that are valid for multiple imaging modalities. This is especially challenging when using immobile methods, such as fMRI, although implementing real-stepping is a possible solution. Real stepping in a supine position, as required by an fMRI, limits the number of balance challenge tasks that can be evaluated, for example to surface perturbations. Although fMRI has a superior spatial resolution as compared to fNIRS, the mobility of the fNIRS hardware provides an advantage in studying the full range of balance challenges. Therefore, future research should investigate paradigms that combine EEG and fNIRS. Simultaneous EEG-NIRS recording systems have been successfully used in language studies, but the combination of electrodes and optodes was not mobile (Wallois et al., 2012). Given the potential of multi-modality neuroimaging within the balance control domain, two main recommendations for future work include:

1. The spatial resolution of fMRI, PET, and SPECT neuroimaging modalities is valuable for elucidating the neural sources involved in balance control. However, future multi-modal studies including one of these modalities should implement an active leg movement paradigm, similar to (Shine et al., 2013a,b; Jaeger et al., 2016), as this more closely resembles real gait as compared to locomotion imagery.
2. Research groups working to improve neuroimaging hardware and software should focus on technical challenges hindering the pairing of fNIRS and EEG modalities. A multi-modal, mobile fNIRS and EEG system could leverage the spatial and temporal resolution of each, providing additional insights into the brain activity involved in balance control tasks.

## Limitations

This review was limited by several factors:

1. The search databases, terminology, and dates directly impact the literature included in this review. Final searches in all databases were performed on September 1, 2016; therefore, this review only includes studies published on or before this date. Additionally, the choice of search terms was based on keywords and terminology in current literature within the neuroimaging and balance control domains. This may have led to exclusion of studies with different terminology or emphasis, including early work on cortical potentials evoked by balance control challenges. Studies by Dietz et al., 1984; Ackermann et al., 1986, for example, characterized the cortical potential occurring after a balance challenge, terming it the perturbation-evoked potential. Several studies included in this review extend the findings of these early papers (Mihara et al., 2007; Adkin et al., 2008; Jacobs et al., 2008; Mochizuki et al., 2008). Therefore, the results of this review are not impacted by the exclusion of these early papers.
2. This review focused only on brain activity during balance control tasks, and did not attempt to analyze muscle activity during these tasks. Electromyographic signals are necessary for motion and, as an indirect indicator of neural activity, are thus involved in maintaining balance control. While correlation between muscle and brain activity may provide additional insights into the mechanisms involved in balance control, analysis of electromyography results was beyond the scope of this review.

## Future work

Further analysis of balance control paradigms suggests that there are significant opportunities for innovation in neuroimaging research within this domain, which include:

1. Researchers utilizing neuroimaging modalities in the balance control domain have a clear opportunity to couple these modalities with sensory and mechanical balance challenges, including experimental manipulation of optical flow, vestibular inputs, and conditions that impair multi-sensory integration.
2. There is evidence that cognitive loading in patients with neurological disorders, such as Parkinson's disease, or in advancing age impacts the ability to main upright stance (Wajda and Sosnoff, 2015) and may increase risk of falls. These populations should continue to be included in future research to elucidate the neural mechanisms governing and potential compensatory mechanisms involved in balance control responses.
3. Movement artifact identification and removal is obviously still an issue to be addressed. Recent studies have revealed high inter-subject variability and the difficulty in completely eliminating gait-related movement artifacts from EEG signals (Castermans et al., 2014; Bradford et al., 2015; Kline et al., 2015; Snyder et al., 2015; Nathan and Contreras-Vidal, 2016). It is clear that future work should focus on improving techniques and methodologies for signal processing of acquired mobile signals.
4. Engineers focused on the development of mobile neuroimaging hardware have an opportunity to include design elements that will dampen the noise user movement introduces into the signal. Additionally, design engineers should remain aware of human factors considerations during the design process, including hardware usability and user satisfaction for all user groups.
5. Lastly, as mentioned by Oliveira et al. (2016), there is a need for benchmarking mobile neuroimaging acquisition systems, specifically in terms of repeatability, signal quality, and user satisfaction of the various hardware and software systems.

The nine recommendations (two for mobile imaging, two for multi-modal imaging, and five for general balance control domain) put forward in this review provide a foundation for future investigation of neuroimaging within the balance control domain. The relatively recent breakthroughs in mobile neuroimaging paradigms have allowed researchers to investigate brain activity during natural human movement (Gramann et al., 2014). However, it is clear that the application of mobile neuroimaging systems in the human balance control domain is still in its early stages. There remains considerable opportunities in identifying neural mechanisms underlying human balance control and the use of these systems in truly mobile, real-world balance challenges.

## Author contributions

All authors contributed equally to this work by conducting the review and drafting the initial manuscript. EW and JT were responsible for writing and editing the current manuscript under supervision of CN and JF who oversaw the project and guided it to completion. All authors discussed the results and implications and commented on the manuscript at all stages.

### Conflict of interest statement

The authors declare that the research was conducted in the absence of any commercial or financial relationships that could be construed as a potential conflict of interest.
